# **Pandemic Influenza Threat and Preparedness** 1

**DOI:** 10.3201/eid1201.050983

**Published:** 2006-01

**Authors:** Anthony S. Fauci

**Affiliations:** * National Institutes of Health, Bethesda, Maryland, USA

**Keywords:** Pandemic, influenza, avian, countermeasure, vaccine, antiviral, bird flu, perspective

## Abstract

The threat of a human influenza pandemic has greatly increased over the past several years with the emergence of highly virulent avian influenza viruses, notably H5N1 viruses, which have infected humans in several Asian and European countries. Previous influenza pandemics have arrived with little or no warning, but the current widespread circulation of H5N1 viruses among avian populations and their potential for increased transmission to humans and other mammalian species may afford us an unprecedented opportunity to prepare for the next pandemic threat. The US Department of Health and Human Services is coordinating a national strategy to respond to an influenza pandemic that involves multiple agencies, including the Centers for Disease Control and Prevention, the Food and Drug Administration, and the National Institutes of Health (NIH). Within NIH, the National Institute of Allergy and Infectious Diseases (NIAID) conducts basic and clinical research to develop new vaccine technologies and antiviral drugs against influenza viruses. We describe recent research progress in preparing for pandemic influenza.

## Abstract 2

New vaccine technologies and antiviral drugs are needed to prepare for the next influenza pandemic.

Since December 2003, H5N1 avian influenza viruses have killed millions of domestic fowl in Southeast Asia (tens of millions more have been culled). It has also infected >130 persons and killed >70 in Vietnam, Thailand, Cambodia, Indonesia, and China (Figure 1) (*1*). If the virus acquires the ability to transmit readily among humans, an influenza pandemic could ensue, with the potential to kill millions of people (*2*). Reports in both the popular press (*3*) and scientific literature (*4**–**7*) have raised alarms in the United States and throughout the world. The prospect of pandemic influenza provides good reason to be concerned. Rather than react in panic, however, we need to determine what can be done now with the knowledge and resources currently available to prevent or minimize the impact of a potential pandemic. At the same time we must ask how we can improve our infrastructure and technology to prepare for future outbreaks.

**Figure 1:**
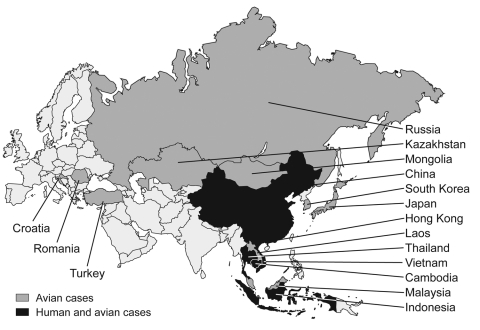
H5N1 cases in Asia, 2004–2005, among birds (dark gray) and humans (black) (*1*). A total of 137 laboratory-confirmed cases, including 70 deaths, occurred. This total includes 22 human cases and 14 deaths in Thailand, 93 human cases and 42 deaths in Vietnam, 4 human cases and 4 deaths in Cambodia, 13 human cases and 8 deaths in Indonesia, and 5 human cases and 2 deaths in China (*1*). A total of 137 laboratory-confirmed cases, including 70 deaths, occurred. This total includes 22 human cases and 14 deaths in Thailand, 93 human cases and 42 deaths in Vietnam, 4 human cases and 4 deaths in Cambodia, 13 human cases and 8 deaths in Indonesia, and 5 human cases and 2 deaths in China.

Unlike seasonal influenza epidemics caused by viruses that mutate in small but important ways from year to year, a process known as antigenic drift, pandemic influenza is caused by a virus that is dramatically different from those that have circulated previously, which can occur through a phenomenon referred to as antigenic shift (*2*). Such viruses can cause pandemics because few people, or none at all, have had prior immunologic exposure to surface proteins of these viruses. In a typical interpandemic influenza season, people may have some residual immunity from exposure to previously circulating influenza strains or from vaccinations (*8*). For example, the predominant circulating influenza virus in the Northern Hemisphere during the 2004–2005 influenza season was an H3N2 virus that had drifted somewhat but was still fundamentally similar to the H3N2 viruses that had circulated in 2003–2004 and previously. Nonetheless, a virus that has undergone antigenic drift can cause illness and death; vaccination provides varying degrees of protection from severe illness and death from influenza complications (*8*). Pandemic influenza, however, can cause a public health crisis because most people would be immunologically naive to the new virus. In addition, the pandemic virus might be inherently more virulent than interpandemic strains. Whereas seasonal influenza rarely threatens the lives of young and otherwise healthy persons, pandemic influenza frequently has exacted a serious toll in healthy, young adults (*2**,**9*).

As of December 2005, outbreaks of H5N1 avian influenza viruses had occurred in domestic poultry populations in at least 16 countries in Asia and eastern Europe (*10*). H5N1 viruses have also have been isolated from wild birds. Disease caused by H5N1 and presence of the virus among thousands of migratory wildfowl have been observed in western China, and more recently, in Kazakhstan, Mongolia, and Croatia, which raises the possibility that H5N1 may be spreading from its stronghold in Southeast Asia through migratory flyways (*11**–**13*). In addition to a growing list of avian species, the virus has infected several mammalian species, including tigers, leopards, and pigs, and transmission among domestic cats has been observed in the laboratory (*14**–**16*). Together, these findings suggest that both the geographic and host ranges of H5N1 viruses are expanding.

The true extent of human H5N1 infections is not precisely known; preliminary reports suggest that the extent of bird-to-human transmission may be more widespread than originally thought (*17*). Thus far, the virus has not acquired the ability to be efficiently transmitted from human to human, although a recent report describes the possible transmission of H5N1 within a family in Thailand (*18*).

The H5N1 avian influenza viruses now circulating may be the most likely candidates for triggering an influenza pandemic because of ongoing reports of new cases in humans (*19*). However, other avian influenza viruses also are being monitored for their potential to infect and cause disease in humans (Figure 2). The H9N2 influenza virus, although not highly pathogenic, has circulated widely among birds in Hong Kong and China; it infected 2 children in 1999 (*20**,**21*) and 1 child in Hong Kong in 2003 (*22*), each of whom recovered. Five additional human infections with H9N2 viruses were reported in the Chinese literature (*23*). Another avian influenza virus, H7N7, is worrisome because it is highly pathogenic in birds and appears to be more readily transmissible from human to human (*24**,**25*). During a large outbreak of highly pathogenic avian influenza in Europe in 2003, an H7N7 virus was detected in at least 86 poultry workers and 3 family members who had no contact with chickens; these persons were treated for conjunctivitis, influenzalike symptoms, or both. A veterinarian who handled infected chickens died of pneumonia and acute respiratory distress (*24**,**25*). With the exception of this fatal case, the H7N7 virus appeared to be relatively benign for humans. Recent reports indicate that an H7 influenza A virus may be circulating among chickens in North Korea (*26*). If a virus such as H5N1 (which is highly pathogenic in humans) were to acquire the genetic capability that enabled the efficient transmissibility observed with H7N7 or human H1N1 or H3N2 influenza viruses, while maintaining most or all of its pathogenic potential, a deadly pandemic could ensue.

**Figure 2:**
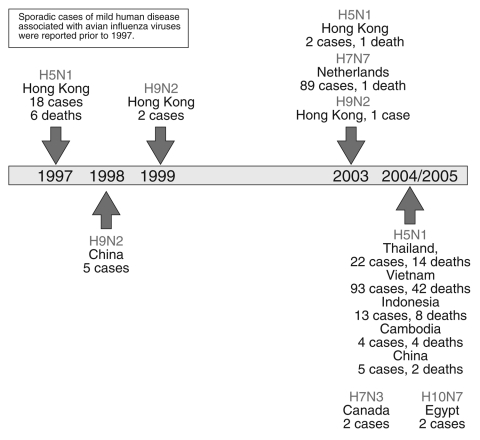
Timeline of documented human infection with avian influenza viruses, 1997–present (*2*). Sporadic cases of mild human disease associated with avian influenza viruses were reported before 1997.

Predicting or preventing the natural events that could facilitate efficient transmission of a pandemic influenza virus among humans is difficult. However, we must be prepared to react quickly and decisively should such an event occur. Critical to the containment of a potential influenza pandemic is diligent surveillance for novel viruses in both human and animal populations using appropriate diagnostics; we must also monitor the viruses for changes that could signal increased virulence or transmissibility. Equally important are the development and production of effective countermeasures, such as vaccines and antiviral drugs (*27*).

## Pandemic Influenza Preparedness

Vaccine development is a critical component of pandemic influenza preparedness. In this regard, the National Institute of Allergy and Infectious Diseases (NIAID) at the National Institutes of Health (NIH) in April 2005 initiated a phase I clinical trial to assess the safety and immunogenicity of different doses of an inactivated H5N1 influenza vaccine manufactured by Sanofi Pasteur (*28*). In this study, 451 healthy adult volunteers were vaccinated at 3 sites in the United States. Preliminary evaluation indicates the vaccine is safe and able to stimulate an immune response that may be protective. The vaccine is currently being tested in the elderly, and testing in children is expected to begin by January 2006.

The H5N1 seed virus used to make this vaccine was developed in a matter of weeks through the use of reverse genetics, whereas the traditional process of genetic reassortment usually requires a longer period of time and is less predictable (*29*). Additional pilot lots of inactivated vaccine are being produced by another manufacturer, Chiron Corporation, and are expected to undergo testing by early 2006.

Chiron also has produced 40,000 doses of an inactivated H9N2 influenza vaccine formulated with and without MF59 adjuvant. Clinical trials to test the safety and immunogenicity of the inactivated H9N2 vaccine are underway, with promising preliminary results. In addition, the US Department of Health and Human Services (HHS) has awarded several contracts to pharmaceutical companies to produce large quantities of bulk H5N1 vaccine as part of the HHS Pandemic Influenza Preparedness Program. These contracts are a critical step toward pandemic influenza preparedness because they pave the way for the manufacturer to commence efficient, large-scale production of any pandemic vaccine if or when it is needed. HHS has also awarded a separate contract to Sanofi Pasteur to accelerate the development of cell culture–based technologies for influenza vaccine production (*28*).

In addition, the intramural research program of NIAID has generated live, attenuated, cold-adapted H9N2 and H5N1 vaccine candidates that have proven protective in mice. The H9N2 vaccine candidate has been tested in a phase I clinical trial, and data are currently being evaluated; clinical evaluation of the H5N1 vaccine is planned for the spring of 2006. Live, attenuated vaccines are especially promising because they generally trigger more rapid and robust immune responses compared with those induced by inactivated vaccines. Live, attenuated vaccines may also offer more cross-reactivity and therefore greater protection against different variants of the same virus (*30*).

The concept of extending vaccine supplies also is being pursued. Research has suggested that delivering vaccines intradermally might allow successful immunization with less antigen (*31**–**33*); clinical trials to compare intramuscular versus intradermal delivery of H5N1 vaccines began in 2005; preliminary safety data showed no adverse effects and immunogenicity data are expected soon. Studies to assess the effect of alum and MF59 adjuvants on inactivated H5N1 vaccine safety and immunogenicity also are anticipated.

Other research efforts are focused on medications to treat influenza infection. Unfortunately, most currently circulating H5N1 influenza viruses are resistant to 2 inexpensive antiinfluenza drugs, rimantadine and amantadine, that target the viral M2 protein. Newer drugs such as oseltamivir phosphate and zanamivir that target the influenza neuraminidase protein appear to be effective against most current H5N1 strains (*34*). HHS and the Centers for Disease Control and Prevention have begun developing a stockpile of antiinfluenza drugs that includes oseltamivir phosphate, zanamivir, and rimantadine for future use should pandemic influenza occur. Numerous other projects are under way to identify novel drug targets and develop compounds that inhibit viral entry, replication, and maturation (*28*).

Underpinning these efforts are basic research studies. For example, NIAID coordinates the Influenza Genome Sequencing Project, a collaborative effort to create complete genetic blueprints of known human and avian influenza viruses. As of December 7, 2005, a total of 559 influenza genome sequences have been made publicly available in GenBank by the NIAID project (*35*). In a separate but related contract awarded to researchers at St. Jude Children's Research Hospital, animal influenza viruses from wild birds, live bird markets, and pigs in Hong Kong and North America are being sequenced, and surveillance has expanded to include additional sites in Asia. The goal of these projects is to rapidly sequence influenza genomes derived from a variety of human and animal sources to enable scientists to understand how the viruses evolve, spread, and cause disease. The long-term goal is improving methods of prevention and treatment.

## The Fragile Vaccine Enterprise

As we develop strategies to prepare for an influenza pandemic, we need to address the overall fragility of the entire vaccine research and manufacturing enterprise (*27**,**36**,**37*). Many pharmaceutical companies are reluctant to enter or remain in the business of manufacturing vaccines. Unpredictable consumer demands and lack of financial incentives make vaccine manufacturing a risky business in today's marketplace. This situation is particularly true with influenza vaccine. Strong collaborations among government, academia, and industry are needed to ensure a reliable vaccine supply. The biomedical research community can help by developing state-of-the-art technologies and sharing them with industry to streamline the manufacturing process and make it more flexible, predictable, and able to adapt to the evolving nature of influenza viruses and other pathogens. Financial and economic incentives, including fair pricing and guaranteed purchase of unsold supplies, regulatory relief, tax incentives, liability protection, and intellectual property considerations, are needed to ensure a steady supply of vaccines (*27**,**36**,**37*). Although the fragility of the vaccine industry cannot be fixed overnight, the process needs to be initiated now to adequately prepare for future pandemics.

## Lessons from Severe Acute Respiratory Syndrome

Recent experience with an outbreak of severe acute respiratory syndrome (SARS) serves as an instructive example in preparing for a potential influenza pandemic (*38**,**39*). In 2002, the deadly respiratory disease emerged and rapidly spread to Canada, Vietnam, Hong Kong, and other sites in China, ultimately resulting in 8,098 cases and 774 deaths. The outbreak, which elicited a classic study in epidemiologic investigation with regard to identifying the point source, tracking the spread, and instituting containment measures, taught us many important lessons. Academic scientists, public health officials, and commercial pharmaceutical companies acted together in an unprecedented way, leading to the development of promising vaccine candidates in record time. The etiologic agent of SARS, a previously unrecognized coronavirus, was identified in March 2003 and sequenced within 2 weeks, and a vaccine candidate was developed by the following March. In December 2004, a clinical trial of a candidate SARS vaccine began at the NIH Vaccine Research Center (*40*).

Because the SARS coronavirus is not as easily transmitted as influenza viruses, we do not know whether the actions that led to the containment of SARS would be as successful if an avian influenza virus acquired the ability to spread efficiently from person to person. However, we have an added advantage in bracing for pandemic influenza that we did not have with SARS. As noted, SARS is caused by a coronavirus that was unknown before the 2003 outbreak. In the current situation, we have identified the H5N1 virus as a likely candidate for triggering a pandemic.

We cannot be certain when the next influenza pandemic will emerge, or even whether it will be caused by H5N1 or an unrelated virus. However, we can be certain that an influenza pandemic eventually will occur. The efforts currently under way to monitor the evolution and spread of H5N1 and other influenza viruses and to develop candidate vaccines and appropriate countermeasures will help in developing the infrastructure and manufacturing capacity that will be required to scale up vaccine and antiviral production when the pandemic occurs.

Because quantities of vaccine and antiviral drugs against a pandemic influenza virus will be limited, deciding beforehand how to best use our resources throughout the world to minimize the impact of pandemic influenza is critical. Global cooperation will be vital. During the SARS epidemic, the World Health Organization created an outstanding network of laboratories and public health agencies from countries around the globe that were indispensable in identifying and ultimately containing the spread of the virus. To adequately address the many research issues surrounding avian influenza and other potential pandemic pathogens, NIAID's Office of Clinical Research is establishing a Southeast Asia Clinical Trials Network to evaluate influenza interventions. This network builds upon existing infrastructure where possible and will be a true partnership between the investigators and the healthcare leadership of the target countries. Such international teamwork is essential as we prepare for an influenza pandemic.

## References

[1] World Health Organization. Confirmed human cases of avian influenza A(H5N1). 2005 [cited 2005 Oct 31]. Available from http://www.who.int/csr/disease/avian_influenza/country/en/

[2] World Health Organization. Avian influenza: assessing the pandemic threat. 2005 [cited 2005 Oct 31]. Available from http://www.who.int/csr/disease/influenza/WHO_CDS_2005_29/en/index.html

[3] Specter M. Nature's bioterrorist. New Yorker. 2005;50–61.

[4] Webby RJ, Webster RG. Are we ready for pandemic influenza? Science. 2003;302:1519–22. 10.1126/science.1090350 14645836

[5] Monto AS. The threat of and an avian influenza pandemic. N Engl J Med. 2005;352:323–5. 10.1056/NEJMp048343 15668220

[6] Stohr K. Avian influenza and pandemics: research needs and opportunities. N Engl J Med. 2005;352:405–7. 10.1056/NEJMe048344 15668221

[7] Institute of Medicine. The threat of pandemic influenza: are we ready? 2004 Nov [cited 2005 Oct 21]. Available from http://www.iom.edu/report.asp?id=23639

[8] Couch RB. An overview of serum antibody responses to influenza virus antigens. Dev Biol (Basel). 2003;115:25–30. 15088772

[9] Reid AH, Taubenberger JK, Fanning TG. The 1918 Spanish influenza: integrating history and biology. Microbes Infect. 2003;3:81–7. 10.1016/S1286-4579(00)01351-4 11226857

[10] World Organization for Animal Health. Update on avian influenza in animals (type 5). 2005 Oct 28 [cited 2005 Oct 31]. Available from http://www.oie.int/downld/AVIAN%20INFLUENZA/A_AI-Asia.htm

[11] Food and Agriculture Organization. AIDE News: AI Bulletin. Update on the avian influenza situation (as of 1/9/2005). Issue no. 33. [cited 2005 Oct 31]. Available from http://www.fao.org/ag/againfo/subjects/documents/ai/AVIbull033.pdf

[12] Chen H, Smith GJ, Zhang SY, Qin K, Wang J, Li KS, Avian flu: H5N1 virus outbreak in migratory water fowl. Nature. 2005;436:191–2. 10.1038/nature03974 16007072

[13] Liu J, Xiao H, Lei F, Zhu Q, Qin K, Zhang X, Highly pathogenic H5N1 influenza virus infection in migratory birds. Science. 2005;309:1206. 10.1126/science.1115273 16000410

[14] Kuiken T, Rimmelzwaan G, van Riel D, van Amerongen G, Baars M, Fouchier R, Avian influenza in cats. Science. 2004;306:241. 10.1126/science.1102287 15345779

[15] Keawcharoen J, Oraveerakul K, Kuiken T, Fouchier RA, Amonsin A, Payungporn S, Avian influenza in tigers and leopards. Emerg Infect Dis. 2004;10:2189–91. 15663858 10.3201/eid1012.040759 PMC3323383

[16] World Health Organization. Avian influenza: H5N1 detected in pigs in China. WHO Communicable Disease Surveillance and Response. 2004 Aug 20 [cited 2005 Oct 21]. Available from http://www.who.int/csr/don/2004_08_20/en/

[17] Olsen SJ, Ungchusak K, Sovann L, Uyeki TM, Dowell SF, Cox NJ, Family clustering of avian influenza A (H5N1). Emerg Infect Dis. 2005;11:1799–801. 16422010 10.3201/eid1111.050646 PMC3367331

[18] Ungchusak K, Auewarakul P, Dowell SF, Kitphati R, Auwanit W, Puthhavathana P, Probable person-to-person transmission of avian influenza A (H5N1). N Engl J Med. 2005;352:333–40. 10.1056/NEJMoa044021 15668219

[19] World Health Organization. Avian influenza: situation updates. 2005 [cited 2005 Oct 21]. Available from http://www.who.int/csr/disease/avian_influenza/updates/en/

[20] Peiris M, Yuen KY, Leung CW, Chan KH, Ip PL, Lai RW, Human infection with influenza H9N2. Lancet. 1999;354:916–7. 10.1016/S0140-6736(99)03311-5 10489954

[21] Choi YK, Ozaki H, Webby RJ, Webster RG, Peiris JS, Poon L, Continuing evolution of H9N2 influenza viruses in southeastern China. J Virol. 2004;78:8609–14. 10.1128/JVI.78.16.8609-8614.2004 15280470 PMC479067

[22] Centers for Disease Control and Prevention. Avian influenza infection in humans. 2005 Oct 17 [cited 2005 Oct 21]. Available from http://www.cdc.gov/flu/avian/gen-info/avian-flu-humans.htm

[23] Guo Y, Li J, Cheng X. Discovery of men infected by avian influenza A H9N2 virus. Zhonghua Shi Yan He Lin Chuang Bing Du Xue Za Zhi. 1999;13:105–8. 12569771

[24] Fouchier RA, Schneeburger PM, Rozendaal FW, Broekman JM, Kemink SA, Munster V, Avian influenza A virus (H7N7) associated with human conjunctivitis and a fatal case of acute respiratory distress syndrome. Proc Natl Acad Sci U S A. 2004;101:1356–61. 10.1073/pnas.0308352100 14745020 PMC337057

[25] Koopmans M, Wilbrink B, Conyn M, Natrop G, van der Nat H, Vennema H, Transmission of H7N7 avian influenza A virus to human beings during a large outbreak in commercial poultry farms in the Netherlands. Lancet. 2004;363:587–93. 10.1016/S0140-6736(04)15589-X 14987882

[26] Food and Agriculture Organization. AIDE News: AI Bulletin. Update on the avian influenza situation (as of 12/05/2005). Issue no. 30. [cited 2005 Oct 21]. Available from http://www.fao.org/ag/againfo/subjects/documents/ai/AVIbull030.pdf

[27] Fauci AS. Race against time. Nature. 2005;435:423–4. 10.1038/435423a 15917781

[28] National Institutes for Allergy and Infectious Diseases. Focus on the flu. 2005 Jun 21 [cited 2005 Oct 21]. Available from http://www2.niaid.nih.gov/Newsroom/FocusOn/Flu04/#resources

[29] Webby RJ, Perez DR, Coleman JS, Guan Y, Knight JH, Govorkova EA, Responsiveness to a pandemic alert: use of reverse genetics for rapid development of influenza vaccines. Lancet. 2004;363:1099–103. 10.1016/S0140-6736(04)15892-3 15064027 PMC7112480

[30] Clements ML, Betts RF, Murphy BR. Advantage of live attenuated cold-adapted influenza A virus over inactivated vaccine for A/Washington/80 (H3N2) wild-type virus infection. Lancet. 1984;1:705–8. 10.1016/S0140-6736(84)92222-0 6143042

[31] La Montagne JR, Fauci AS. Intradermal influenza vaccination: can less be more? N Engl J Med. 2004;351:2330–2. 10.1056/NEJMe048314 15525715

[32] Belshe RB, Newman FK, Cannon J, Duane C, Treanor J, van Hoecke C, Serum antibody responses after intradermal vaccination against influenza. N Engl J Med. 2004;351:2286–94. 10.1056/NEJMoa043555 15525713

[33] Kenney RT, Frech SA, Muenz LR, Villar CP, Glenn GM. Dose sparing with intradermal injection of influenza vaccine. N Engl J Med. 2004;351:2295–301. 10.1056/NEJMoa043540 15525714

[34] Ward P, Small I, Smith J, Suter P, Dutkowski R. Oseltamivir (Tamiflu) and its potential for use in the event of an influenza pandemic. J Antimicrob Chemother. 2005;55(Suppl 1):i5–21. 10.1093/jac/dki018 15709056

[35] National Center for Biotechnology Information. Influenza virus resource. 2005 Jun 23 [cited 2005 Oct 31]. Available from http://www.ncbi.nlm.nih.gov/genomes/FLU/FLU.html

[36] Sloan FA, Berman S, Rosenbaum S, Chalk RA, Giffin RB. The fragility of the U.S. vaccine supply. N Engl J Med. 2004;351:2443–7. 10.1056/NEJMsb033394 15575064

[37] Poland GA, Marcuse EK. Vaccine availability in the US: problems and solutions. Nat Immunol. 2004;5:1195–8. 10.1038/ni1204-1195 15549115

[38] Muller MP, McGeer A, Straus SE, Hawryluck L, Gold WL. Clinical trials and novel pathogens: lessons learned from SARS. Emerg Infect Dis. 2004;10:389–94. 15109402 10.3201/eid1003.030702 PMC3322802

[39] Finlay BB, See RH, Brunham RC. Rapid response research to emerging infectious diseases. Nat Rev Microbiol. 2004;2:602–7. 10.1038/nrmicro930 15197395 PMC7097457

[40] National Institute of Allergy and Infectious Diseases. First US SARS vaccine trial opens at NIH. 2004 Dec 13 [cited 2005 Oct 21]. Available from http://www2.niaid.nih.gov/newsroom/releases/sarstrial.htm

